# Supplementary material for: Molecular insights into genome-wide association studies of chronic kidney disease-defining traits
Source: Nat Commun. 2018 Nov 22;9:4800. doi: 10.1038/s41467-018-07260-4 (PMC6250666; doi:10.1038/s41467-018-07260-4)
Supplement: Supplementary file 1 — Description of Additional Supplementary Files [file 41467_2018_7260_MOESM1_ESM.pdf]

Description of Additional Supplementary Files

Supplementary Data 1. Best transcriptionally active single nucleotide variant for each kidney eGene identified in the analysis of TRANSLATE Study and TCGA.

Supplementary Data 2. Summary information for 75 eGenes showing kidney-enrichment in Human Protein Atlas (www.proteinatlas.org).

Supplementary Data 3. Variants showing significant genome-wide associations with chronic kidney disease defining traits.

Supplementary Data 4. Transcriptionally active single nucleotide variants showing significant genome-wide associations with chronic kidney disease defining traits and their partner renal eGenes.

Supplementary Data 5. Characteristics of 35 expression partner genes (eGenes) for variants associated with chronic kidney disease defining traits in genome-wide association studies.

Supplementary Data 6. Transcriptionally active single nucleotide variants associated with chronic kidney disease defining traits in genome-wide association studies whose kidney expression partners (renal eGenes) are different from the nearest genes (by proximity).

Supplementary Data 7. Renal expression partner genes (eGenes) of transcriptionally active single nucleotide variants associated with chronic kidney disease defining traits in genome-wide association studies and their target renal cell type in single-cell RNA-sequencing of human kidneys based on Gillies et al.

Supplementary Data 8. Association between kidney diseases and renal expression partner genes (eGenes) for transcriptionally active single nucleotide variants associated with chronic kidney disease defining traits in genome-wide association studies (CKD-dt GWAS eSNPs) – case-control studies from Nephroseq.

Supplementary Data 9. Association between estimated glomerular filtration rate and renal expression partner genes (eGenes) of transcriptionally active single nucleotide variants associated with chronic kidney disease defining traits in genome-wide association studies (CKD-dt GWAS eSNPs) – Nephroseq-based analysis.

Supplementary Data 10. The pairs of transcriptionally active single nucleotide variants identified in genome wide association studies of chronic kidney disease defining traits (CKD-dt GWAS eSNPs) and their renal expression partner genes (eGenes) identified in the kidney (TRANSLATE Study and TCGA) or non-renal tissues (Genotype-Tissue Expression project) – single tissue *cis*-expression quantitative trait loci analysis.

Supplementary Data 11. Overview of transcriptionally active single nucleotide variants associated with chronic kidney disease defining traits in genome-wide association studies (CKD-dt GWAS eSNPs) – single and multi-tissue analyses.

Supplementary Data 12. All pairs of transcriptionally active single nucleotide variants identified in genome wide association studies of chronic kidney disease defining traits (CKD-dt GWAS eSNPs) and their renal expression partner genes (eGenes) identified in non-renal tissues (Genotype-Tissue Expression project) – multiple tissue *cis*-expression quantitative trait loci analysis.

Supplementary Data 13. Pleiotropy analysis using PhenoScanner on kidney-specific and ubiquitous transcriptionally active single nucleotide variants associated with chronic kidney disease defining traits in genome-wide association studies.

Supplementary Data 14. Functional annotations for 117 sentinel variants associated with chronic kidney disease defining traits in genome-wide association studies.

Supplementary Data 15. Functional annotations for 117 sentinel variants and their 2,672 proxies associated with chronic kidney disease-defining traits in genome-wide association studies.

Supplementary Data 16. Summary results for penalised weighted median, robust inverse variance weighted, robust MR-Egger regression as well as pleiotropy and heterogeneity test for 7 kidney eGenes implicated by colocalisation analysis.
