# Supplementary material for: Molecular insights into genome-wide association studies of chronic kidney disease-defining traits
Source: Nat Commun. 2018 Nov 22;9:4800. doi: 10.1038/s41467-018-07260-4 (PMC6250666; doi:10.1038/s41467-018-07260-4)
Supplement: Supplementary file 2 — Supplementary Information [file 41467_2018_7260_MOESM2_ESM.pdf]

Supplementary information

# Molecular insights into genome-wide association studies of chronic kidney disease-defining traits

Xu et al.

**Supplementary table 1.** General characteristics of the TRANSLATE study and TCGA.

| | TRANSLATE | TCGA |
|---|---|---|
| n | 180 | 100 |
| Age (years) | 61.3 (10.7) | 61.2 (13.2) |
| Men | 99 (55%) | 70 (70%) |
| Ethnicity | white European | white European |
| BMI ($kg/m^2$) | 27.8 (5.3) | NA |
| eGFR ($ml/min/1.73m^2$) | 76.2 (20.1) | NA |

Data are counts and percenatges or means and standard deviations where appropriate, n - number of individuals, BMI - body mass index, eGFR - estimated glomerular filtration rate. In TRANSLATE Study circulating concentrations of creatinine, were used to calculate eGFR based on the EPI equation [Levey, A. S. et al. A new equation to estimate glomerular filtration rate. Ann. Intern. Med. 150, 604–612 (2009)].

**Supplementary table 2**. Number of genome-wide association study-pleiotropic or non-pleiotropic eSNPs (single nucleotide variants transcriptionally active in the kidney) stratified upon kidney-specificity (tissue-specific versus ubiquitous).

| | **Kidney-specific** | **Ubiquitous** |
|---|---|---|
| Non-pleiotropy | 5 | 16 |
| Pleiotropy | 1 | 31 |
| Fisher's exact test P-value: 0.0307 | | |

**Supplementary table 3**. Colocalisation analyses.

| ID | CKD-dt GWAS SNP dbSNP rsID | CKD-dt GWAS SNP Position | CKD-dt GWAS SNP Chr | Renal eGene | Renal eGene symbol | RTC score | Coloc-alised |
|---|---|---|---|---|---|---|---|
| 1 | rs12124078 | 15869899 | 1 | ENSG00000132906 | *CASP9* | 0.93 | Yes |
| 6 | rs2049805 | 155194980 | 1 | ENSG00000160766 | *GBAP1* | 0.77 | No |
| 6 | rs2049805 | 155194980 | 1 | ENSG00000185499 | *MUC1* | 0.97 | Yes |
| 7 | rs6677604 | 196686918 | 1 | ENSG00000244414 | *CFHR1* | 0.55 | No |
| 9 | rs2802729 | 243501763 | 1 | ENSG00000054282 | *SDCCAG8* | NA | No |
| 13 | rs10206899 | 73900900 | 2 | ENSG00000204872 | *NAT8B* | 0.99 | Yes |
| 24 | rs347685 | 141807137 | 3 | ENSG00000114126 | *TFDP2* | 0.99 | Yes |
| 31 | rs228611 | 103561709 | 4 | ENSG00000246560 | *RP11-10L12.4* | 0.90 | Yes |
| 34 | rs12654812 | 176794191 | 5 | ENSG00000169220 | *RGS14* | 0.43 | No |
| 35 | rs7759001 | 27341409 | 6 | ENSG00000124613 | *ZNF391* | 0.82 | No |
| 36 | rs2523946 | 29941943 | 6 | ENSG00000227262 | *HCG4B* | 0.32 | No |
| 52 | rs3127573 | 160681393 | 6 | ENSG00000122194 | *PLG* | NA | No |
| 67 | rs4077515 | 139266496 | 9 | ENSG00000213221 | *DNLZ* | 0.86 | No |
| 70 | rs10994860 | 52645424 | 10 | ENSG00000204147 | *ASAH2B* | 0.19 | No |
| 73 | rs2074038 | 44087989 | 11 | ENSG00000110455 | *ACCS* | 0.99 | Yes |
| 81 | rs626277 | 72347696 | 13 | ENSG00000276644 | *DACH1* | 0.55 | No |
| 82 | rs2928148 | 41401550 | 15 | ENSG00000187446 | *CHP1* | NA | No |
| 83 | rs2467853 | 45698793 | 15 | ENSG00000259520 | *CTD-2651B20.3* | 0.64 | No |
| 83 | rs2467853 | 45698793 | 15 | ENSG00000171763 | *SPATA5L1* | 0.48 | No |
| 91 | rs164748 | 89708292 | 16 | ENSG00000131165 | *CHMP1A* | 0.62 | No |
| 91 | rs164748 | 89708292 | 16 | ENSG00000015413 | *DPEP1* | 0.37 | No |
| 91 | rs164748 | 89708292 | 16 | ENSG00000204991 | *SPIRE2* | 0.92 | Yes |
| 93 | rs4227 | 7491177 | 17 | ENSG00000141504 | *SAT2* | NA | No |
| 95 | rs11078903 | 37631924 | 17 | ENSG00000161395 | *PGAP3* | 0.51 | No |
| 101 | rs12458009 | 59350507 | 18 | ENSG00000176641 | *RNF152* | 0.37 | No |
| 107 | rs13038305 | 23610262 | 20 | ENSG00000173335 | *CST9* | 0.81 | No |

ID - internal identifier for each of 117 variants, RTC score - regulatory trait concordance score, NA - the GWAS SNP is located in hotspot hence RTC score could not be obtained, CKD-dt - chronic kidney disease and its defining traits, GWAS - genome-wide association study, SNP - single nucleotide polymorphism, Chr - Chromosome, eGene - gene whose expression is associated with at least one SNPs

**Supplementary table 4**. Genomic location, alleles and combined annotation dependant depletion scores for all single nucleotide polymorphisms in the rs2049805 locus.

| dbSNP rsID | GRCh37 chromosome | GRCh37 position | Reference allele | Alternate allele | CADD raw score | Phred-scaled CADD score |
|---|---|---|---|---|---|---|
| rs4072037 | 1 | 155162067 | C | T | 1.284 | 12.19 |
| rs12411216 | 1 | 155164480 | C | A | 0.991 | 10.61 |
| rs2990245 | 1 | 155197462 | C | T | 0.759 | 9.205 |
| rs497829 | 1 | 155193532 | C | G | 0.554 | 7.832 |
| rs2075570 | 1 | 155182164 | C | T | 0.400 | 6.611 |
| rs2974930 | 1 | 155196717 | A | G | 0.288 | 5.576 |
| rs2049805 | 1 | 155194980 | T | C | 0.212 | 4.801 |
| rs914615 | 1 | 155175892 | A | G | 0.105 | 3.664 |
| rs2974929 | 1 | 155197268 | T | C | 0.061 | 3.201 |
| rs2974931 | 1 | 155195215 | C | G | 0.037 | 2.955 |
| rs2066981 | 1 | 155172379 | G | A | -0.154 | 1.359 |
| rs2974937 | 1 | 155168849 | C | T | -0.158 | 1.335 |
| rs2990220 | 1 | 155190254 | T | A | -0.265 | 0.796 |
| rs2974935 | 1 | 155181843 | G | A | -0.378 | 0.438 |
| rs28445596 | 1 | 155186729 | T | C | -0.463 | 0.271 |
| rs370545 | 1 | 155175390 | A | G | -0.475 | 0.253 |

dbSNP rsID - single nucleotide polymorphism database idnetifier, CADD - combined annotation dependant depletion

**Supplementary table 5**. Assocation between 6 CpG probes proximal to MUC1 promoter and rs12411216 genotype in TRANSLATE study.

| Probe ID | Beta | Standard error | T-statistic | P-value |
|---|---|---|---|---|
| cg03425468 | 0.04 | 0.04 | 1.22 | 0.2248 |
| cg07945002 | 0.01 | 0.03 | 0.38 | 0.7073 |
| cg12678006 | -0.01 | 0.02 | -0.45 | 0.6511 |
| cg09417889 | 0.05 | 0.04 | 1.39 | 0.1677 |
| cg15434337 | 0.02 | 0.07 | 0.29 | 0.7694 |
| cg27538026 | 0.00 | 0.06 | -0.03 | 0.9774 |

Beta - linear regression coefficient, SE - standard error, P-value - level of statistical significance

**Supplementary table 6.** Association between 6 CpG probes proximal to MUC1 promoter and renal expression of MUC1 - TRANSLATE study.

| Probe ID | Beta | SE | T-statistic | P-value |
|---|---|---|---|---|
| cg03425468 | -57.52 | 63.33 | -0.91 | 0.3665 |
| cg07945002 | -114.68 | 74.61 | -1.54 | 0.1282 |
| cg12678006 | 49.28 | 96.64 | 0.51 | 0.6115 |
| cg09417889 | -80.78 | 57.59 | -1.40 | 0.1645 |
| cg15434337 | -47.11 | 31.97 | -1.47 | 0.1445 |
| cg27538026 | -53.67 | 35.46 | -1.51 | 0.1341 |

Beta - linear regression coefficient, SE - standard error, P-value - level of statistical significance

**Supplementary table 7.** Average renal expression of MUC1 mRNA isoforms in TRANSLATE study and TCGA. Transcripts are ordered from the most abundant (top) to least abundant (bottom).

| Ensembl MUC1 mRNA symbol | Mean expression (transcripts per million) |
|---|---|
| ENST00000485118 | 42.78 |
| ENST00000612778-as | 36.79 |
| ENST00000612778 | 23.15 |
| ENST00000620103 | 14.98 |
| ENST00000462317 | 10.08 |
| ENST00000468978 | 6.05 |
| ENST00000368392 | 2.55 |
| ENST00000368390 | 1.47 |
| ENST00000368393 | 0.66 |
| ENST00000614519 | 0.52 |

**Supplementary table 8.** Causality between renal expression of total *MUC1* and its mRNA isoforms and estimated glomerular filtration rate - robust inverse variance weighted method, Mendelian randomisation-Egger regression (pleiotropy and heterogeneity test).

| MUC1 isoform | MUC1 isoform symbol | Estimate-IVW-robust | P-value-IVW-robust | P-value-pleiotropy | P-value-heter |
|---|---|---|---|---|---|
| ENST00000612778-as | *MUC1-as* | -0.003 | 2.05E-10 | 0.655 | 0.552 |
| ENST00000620103 | *MUC1-001* | -0.004 | 1.91E-08 | 0.740 | 0.514 |
| ENSG00000185499 | *MUC1* | -0.014 | 1.14E-07 | 0.701 | 0.803 |
| ENST00000368390 | *MUC1-014* | -0.004 | 7.92E-07 | 0.560 | 0.677 |
| ENST00000612778 | *MUC1-207* | 0.002 | 3.84E-05 | 1.000 | 0.383 |
| ENST00000368392 | *MUC1-002* | 0.005 | 3.65E-03 | 0.174 | 0.253 |

as - alternative splicing, IVW - inverse variance weighted method, Estimate-IVW-robust - correlation coefficient from robust IVW method, heter - heterogeneity

**Supplementary table 9.** Post-imputation genotype quality control applied to imputed variants in the TRANSLATE study and TCGA.

| | **TRANSLATE** | **TCGA** |
|---|---|---|
| Imputed variants | 47,100,201 | 47,101,134 |
| Variants mapping to the same genomic position | 474,948 | 474,948 |
| MAF< 5% | 39,590,910 | 39,448,754 |
| R2<0.40 | 35,266,275 | 34,907,262 |
| Non-SNPs | 2,955,459 | 2,955,459 |
| HWE<1x10E-6 | 21 | 395 |
| Final number of SNPs | 5,760,291 | 5,892,571 |

SNP - single nucleotide polymorphism, MAF – minor allele frequency, R2 – imputation coefficient, HWE – Hardy-Weinberg equilibrium.
